# Arenavirus-Based Vectors Generate Robust SIV Immunity in Non-Human Primates

**DOI:** 10.3390/vaccines12070735

**Published:** 2024-07-02

**Authors:** Bhawna Sharma, Elena Bekerman, Hoa Truong, Johnny Lee, Maria Gamez-Guerrero, Archana Boopathy, Rohit Mital, Katell Bidet Huang, Sarah Ahmadi-Erber, Raphaela Wimmer, Sophie Schulha, Henning Lauterbach, Klaus Orlinger, Silpa Suthram, Mark G. Lewis, Wade Blair, Tariro Makadzange, Romas Geleziunas, Jeffrey P. Murry, Sarah Schmidt

**Affiliations:** 1 Gilead Sciences, Inc., Foster City, CA 94404, USA; elena.bekerman@gilead.com (E.B.); hoa.truong@gilead.com (H.T.); johnny.lee@gilead.com (J.L.); maria.gamez@gilead.com (M.G.-G.); archana.boopathy@gilead.com (A.B.); shivarohit@gmail.com (R.M.); silpa.suthram@gilead.com (S.S.); wsblair.01@gmail.com (W.B.); tariro.makadzange@crmgresearch.com (T.M.); romas.geleziunas@gilead.com (R.G.); jeff.murry@gilead.com (J.P.M.); 2 Hookipa Pharma Inc., New York, NY 10018, USA; katell.huang@hookipapharma.com (K.B.H.); sarah.ahmadi-erber@hookipapharma.com (S.A.-E.); raphaela.wimmer@hookipapharma.com (R.W.); sophie.schulha@hookipapharma.com (S.S.); henning.lauterbach@hookipapharma.com (H.L.); klaus.orlinger@hookipapharma.com (K.O.); 3 Bioqual, Inc., Rockville, MD 20850, USA; mlewis@bioqual.com

**Keywords:** arenavirus-based vectors, T-cell response, HIV, replicating vector, non-replicating vector, T-cell mediated immunity, B-cell mediated immunity, therapeutic vaccine

## Abstract

Arenavirus-based vectors are being investigated as therapeutic vaccine candidates with the potential to elicit robust CD8 T-cell responses. We compared the immunogenicity of replicating (artPICV and artLCMV) and non-replicating (rPICV and rLCMV) arenavirus-based vectors expressing simian immunodeficiency virus (SIV) Gag and Envelope (Env) immunogens in treatment-naïve non-human primates. Heterologous regimens with non-replicating and replicating vectors elicited more robust SIV IFN-γ responses than a homologous regimen, and replicating vectors elicited significantly higher cellular immunogenicity than non-replicating vectors. The heterologous regimen elicited high anti-Env antibody titers when administered intravenously, with replicating vectors inducing significantly higher titers than non-replicating vectors. Intramuscular immunization resulted in more durable antibody responses than intravenous immunization for both vector platforms, with no difference between the replicating and non-replicating vectors. Overall, both replicating and non-replicating arenavirus vectors generated robust T- and B-cell-mediated immunity to SIV antigens in treatment-naïve non-human primates, supporting further evaluation of these vectors in a clinical setting for HIV therapy.

## 1. Introduction

HIV-cure research aims to induce long-term HIV control in the absence of antiretroviral therapy (ART) to ultimately eliminate HIV. This is challenging because HIV integrates into cellular DNA after infection, establishing a latent reservoir that is refractory to antiretroviral therapy. ART successfully blocks viral replication but does not eliminate viral reservoirs that reside in lymphatic tissues. These reservoirs typically reseed infection if treatment is interrupted. Potent HIV-specific CD8 T cells can mediate natural HIV control [1,2], suggesting that vaccines that effectively enhance HIV-specific CD8 T cells could also induce HIV control. Consistent with this, a recent trial with a vaccine using the HIVACAT T-cell immunogen (HTI) was associated with prolonged time off ART in post hoc analyses [3]. This virologic control was positively correlated with vaccine-induced HTI-specific T-cell responses. Humoral immunity may also be important for controlling HIV, as envelope binding non-neutralizing antibodies correlated with protection in RV144 vaccine trials [4]. Although none of the HIV vaccines developed to date has been able to induce long-term HIV control, there are multiple avenues for potential HIV vaccine improvement. One such area is the delivery platform for the vaccine immunogen. Recent advancements in COVID-19 vaccines underscored the utility of mRNA–lipid nanoparticle (LNP) delivery in generating robust humoral immunity. The mRNA-LNP strategy has shown some promise in inducing humoral immunity in non-human primate (NHP) models, where it elicited tier-2 HIV-neutralizing antibodies [5]. Similarly, adenoviral vectors have elicited robust cellular and humoral immunity in various disease models, including simian immunodeficiency virus (SIV) and HIV [6,7]. However, adenoviral vectors are limited by pre-existing immunity and the development of anti-vector immunity after immunization.

Lymphocytic choriomeningitis virus (LCMV) and Pichinde virus (PICV) are arenaviruses that induce robust immune responses but rarely cause infection in humans. LCMV and PICV naturally infect and activate antigen-presenting cells (APCs) such as dendritic cells and macrophages, stimulating CD8 T-cell immunity through direct antigen presentation with co-stimulation [8,9]. Replicating LCMV (artLCMV) vectors also infect lymphoid tissue stroma cells, inducing the alarmin IL-33 and potent cytotoxic effector CD8 T lymphocytes [10]. Pre-existing immunity to these vectors is rare in humans, with reported rates of antibodies to LCMV below 5% [11,12,13,14,15]. The glycoproteins of LCMV and PICV are highly glycosylated, minimizing the induction of neutralizing antibodies against these viruses. Previous studies in mice and cynomolgus macaques receiving a homologous prime–boost regimen with non-replicating recombinant LCMV (rLCMV) showed increasing SIV-specific T- and B-cell responses after each immunization [16]. Consistent with this, a phase 1 clinical trial evaluating the safety and immunogenicity of a rLCMV vector resulted in induction of robust CMV-specific CD8 T cells and no vector-neutralizing antibody induction [17]. Replicating arenavirus vector-based vaccines (artArena) also induced robust CD8 T-cell responses after vaccination with HPV16 immunogens [18]. These results suggest that arenavirus-based vectors could be repeatedly used to generate robust T- and B-cell responses for durable HIV-specific immunity.

In the present study, we investigated whether heterologous regimens induced greater SIV immunity than a homologous regimen. We compared the immunogenicity of replicating artArena and non-replicating (rArena) arenavirus-based (LCMV and PICV) vectors, using either intravenous (IV) or intramuscular (IM) injection routes in NHPs. We assessed the magnitude of the SIV-specific ELISpot response, the breadth of the T-cell response, and the Env-specific antibody generation after immunization.

## 2. Materials and Methods

### 2.1. Vector Generation and Titration

Non-replicating and replicating arenavirus vectors used in the study are based on old-world LCMV clone 13 (with its glycoprotein [GP] from strain WE) and new-world PICV strain p18 arenaviruses. The non-replicating vectors, rLCMV and rPICV, are bi-segmented vectors that harbor one large segment (L-segment) and one small segment (S-segment). The GP open-reading frame (ORF) was replaced with SIV_smE543_ Gag or Env, resulting in a replication-deficient virus. 

To generate replication-competent attenuated artArena vectors (artLCMV and artPICV), arenavirus vector genomes were modified from their respective bi-segmented parental arenavirus to include one L-segment and two S-segments.

**Figure 1:**
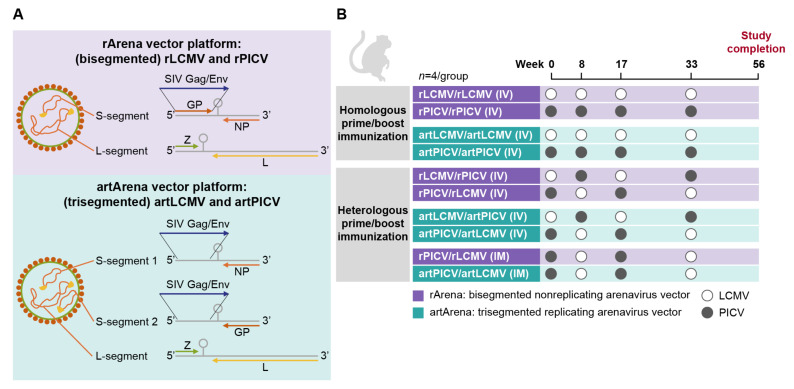
Vector constructs (**A**) and immunogenicity study design (**B**). Schematic diagram of constructs: rArena vector platform (trisegmented; A, upper panel) and artArena vector platform (trisegemented; A, lower). Heterologous LCMV and PICV vectors for prime vs. boost yielded comparable magnitude ELISpot responses by IV route; thus, both rLCMV/rPICV and rPICV/rLCMV and both artLCMV/artPICV and artPICV/artLCMV group responses were combined into a single group (each *n* = 8). Homologous vaccination refers to when prime and boost were administered with the same vector delivering identical immunogen. Heterologous vaccination refers to when two different vectors of the same platform were used to deliver identical immunogen.

Genetically engineered viruses with an artificial genome organization that prevents the occurrence of recombination-based reversion were generated by artificially placing the GP-ORF in a non-natural position under 3′UTR control (instead of 5′UTR). SIV_smE543_ transgenes Gag and Env were inserted under 5′UTR control (Figure 1A). The artificial genome arrangement and the less-efficient packaging of all three genomic segments contribute to replication attenuation, as described previously [19]. 

Both rArena and artArena vectors were produced by transient transfection of LCMV GP-expressing cells with two expression plasmids (encoding the respective LCMV or PICV nucleoprotein [NP] and polymerase L) and plasmids encoding the viral genomes (S- and L-genome) [20]. Newly generated vectors were titrated by focus-forming assays and further passaged on suspension HEK293 cells to generate fetal calf serum-free vector stock material [8,10]. Vector-containing supernatant was harvested, titrated, and analyzed for stable transgene insertion and growth properties.

### 2.2. Study Design and Immunization

NHP studies evaluating the safety and immunogenicity of rArena vectors (rLCMV and rPICV) and artArena vectors (artLCMV and artPICV) were conducted at Bioqual, Inc. (Rockville, MD, USA). Four treatment-naïve Indian-origin rhesus macaques per group were immunized on weeks 0, 8, 17, and 33 IV (groups 1–4) or IM with 1 × 10^7^ FFU of rLCMV and rPICV or 1 × 10^6^ RCV FFU of artLCMV and artPICV vector vaccine, encoding SIV_smE543_Gag or SIV_smE543_Env antigens. The SIVsmE543 strain was selected based on previous studies where SIVsmE543 antigen was evaluated with Ad26/MVA vaccine. SIVSmE543 antigen immunization showed protection [6] and control [21] against heterologous SIV viruses. A 1:1 mixture of vectors expressing SIV_smE543_Gag and SIV_smE543_Env antigens was produced before performing injections (Figure 1). A homologous regimen was performed with either rLCMV, artLCMV or rPICV, artPICV vector alone for a total of four doses. Heterologous immunizations were administered as four alternating doses, starting with LCMV or PICV vectors for a total of four groups: rLCMV/rPICV, rPICV/rLCMV, artLCMV/artPICV, and artPICV/artLCMV (Figure 1B). Homologous immunization was administered IV, whereas heterologous immunizations were administered IV or IM for rPICV/rLCMV and artPICV/artLCMV groups. 

### 2.3. IFN-γ ELISpot

Immune responses to SIV antigens were measured ex vivo by IFN-γ ELISpot analysis upon restimulation of frozen (IV groups up to 10 weeks) or fresh peripheral blood mononuclear cells (PBMCs) with respective peptide pools of Gag and Env at baseline and multiple post-immunization timepoints. Breadth of cellular response was evaluated by ELISpot to 12 Gag and 16 Env peptide subpools comprising 10 peptides each at 2 weeks after IM administration of the fourth dose of rPICV/rLCMV or artPICV/artLCMV regimens. ELISpot was performed using the monkey IFN-γ ELISpot kit from Mabtech (cat# 3421M-4HPW-10) per manufacturer’s recommendations. Briefly, pre-coated 96-well plates provided with the kit were washed four times with PBS (phosphate-buffered saline), followed by the addition of SIV_smE543_Gag or Env peptides (antigens); NP-LCMV or NP-PICV peptides were resuspended in assay media (CTL-Test Medium + 1% L-glutamine) at a final concentration of 2 μg/mL. Then, 10 μg/mL PHA (phytohemagglutinin) was used as a positive control for each sample; assay medium alone served as a negative control. Plates were incubated for 30 min at 37 °C, followed by addition of 100 μL of PBMCs resuspended at 2 × 10^6^ cells/mL in assay media. After overnight incubation at 37 °C, plates were washed with PBS. Then, 100 μL of biotinylated antibody was diluted at 1 μg/mL in 5% FBS in PBS and incubated for 2 h at room temperature (RT), followed by a washing and addition of streptavidin–HRP for 1 h at RT. Plates were developed with Vector Novared substrate per the manufacturer’s protocol (Vector Laboratories, Newark, CA, USA). Once the plates were dried, spots were scanned and counted on an ImmunoSpot analyzer (CTL, S6 Ultimate M2; ImmunoSpot, Cleveland, OH, USA)). Gag- and Env-specific IFN-γ SFUs were calculated for 1 × 10^6^ cells and the magnitude of responses (antigen-specific responses minus 1× background) and graphed as median plus IQR. For Gag- and Env-specific breadth, responses were defined as >3× background signal. Data were plotted as median ± IQR.

### 2.4. Envelope-Binding Antibodies

SIV envelope-binding antibodies were detected against autologous (SIV_sme543_) and heterologous (SIV_smE660_ and SIV_mac251_) SIV strains by ELISA. SIV-gp120 recombinant proteins (Immune Technology Corp., New York, NY, USA) were diluted to 2 μg/mL in sodium bicarbonate buffer at pH 9.4. Then, 25 μL was added to each well of a clear Thermo/Nunc MaxiSorp 384-well assay plate and incubated overnight at 4 °C. Plates were then washed three times with PBS–Tween at pH 7.4 + 0.05% Tween 20 (PBST) using a Biotek 405 plate washer and then blocked for 1 h at RT with 75 μL of the blocking buffer (1% goat serum + DPBS pH 7.4 + 5% skim milk). After blocking, three-fold serially diluted, heat-inactivated sera from vaccinated NHPs were added and incubated in the plate for 1 h at 4 °C. Sera from naïve NHPs were used as a negative control. Plates were washed and incubated for 30 min with 25 uL of goat anti-monkey IgG-HRP secondary antibody (Novus Biologicals, Centennial, CO, USA, NB7215), followed by detection with TMB substrate. Reaction was stopped with 0.16 M sulfuric acid. Plates were read on Envision at 450 nm, and end-point titers were reported as previously described [19]. 

### 2.5. Multiparametric Flow Cytometry

Gag- and Env-specific T-cell polyfunctionality was determined 2 weeks after the fourth vaccination. Fresh (post-vaccination) or frozen (baseline) PBMCs were resuspended in RPMI media supplemented with 10% FBS. Then, 0.5–1 × 10^6^ PBMCs were plated in a 96-well V-bottom plate and treated with DMSO (equal volume to pepmix in media), 2 μg of Gag pepmix, pool of 120 Gag peptides (JPT Peptide Technologies, Berlin, Germany), 2 μg of Env peptide pepmix, pool of 160 Env peptides (JPT Peptide Technologies), and PMA (5 μg/mL) + ionomycin (1 μM) for 1 h at 37 °C in the incubator. After 1 h, Golgi plug and Golgi stop (BD Biosciences, Franklin Lakes, NJ, USA) were added to the cells, and plates were incubated for an additional 12–14 h at 37 °C. After incubation, plates were centrifuged, and cells were washed with PBS and stained with AmCyan dye (live/dead stain, Thermo Scientific, Waltham, MA, USA). After Fc receptor blocking with human TruStain FcX (BioLegend, San Diego, CA, USA), surface staining was performed with anti-CD3 AF700, anti-CD4 BV605, anti-CD8 BV650, anti-CD45RA-PECy7, anti-CD27-BV711, and anti-CCR7-BV785 for 30 min at RT. After two washes with 2% FBS in PBS, cells were fixed with fixation buffer (BD Biosciences) and stained for intracellular markers with anti-human anti-IFN-γ PE-CF594, anti-IL2 PE, and anti-TNF-α PerCPCy5.5 antibodies for 30 min at RT. Cells were washed and resuspended in 2% FBS in PBS for flow data acquisition on BD LSRFortessa. Data were analyzed using FlowJo v.10 and plotted using GraphPad Prism 8.1.2.

### 2.6. LCMV-Neutralizing Antibodies

In a 384-well tissue culture-treated clear-bottom plate, ARPE-19 cells were seeded at 10,000 cells in 40 µL per well with assay media (2% FBS, 1% PS, and 1% glutamine) at 37 °C overnight. The next day, sera samples from vaccinated NHPs were heat-inactivated at 56 °C for 1 h before an eight-point, three-fold serial dilution. An equal volume of rLCMV-GFP green fluorescent protein) vector (Hookipa Pharma, New York, NY, USA) was then added to the diluted sera samples to achieve a final 10,000 PFU/well of VV1 GFP virus. Sera samples (in duplicate for each animal) and LCMV-GFP vector were incubated for 90 min at 37 °C in 5% CO_2_ before infecting the cells. Plated ARPE-19 cells were infected by transferring 40 µL of the sera samples and LCMV-GFP vector, and the cells were incubated for 24 h at 37 °C in 5% CO_2_ before measuring the reduction in the GFP signal. At the end of the 24-h incubation, the medium was removed using a plate washer, without disturbing the cell monolayer. Cells were washed once with 1× PBS. Cells were then fixed with 4% PFA and stained with DAPI (1:1000 dilution) for 30 min at room temperature. Lastly, assay plates were washed three times with 1× PBS before imaging using the Cellomics imager (Thermo Scientific # Cell Insight CX7). Viral vector neutralization results were measured via the reduction of GFP signal and reported as the ID_50_ (inhibitory dose) titers for the sample evaluated. ID_50_ values were calculated from the fit of the dose–response curves to a four-parameter equation. All ID_50_ values represent geometric mean values of a minimum of two determinations. A 1:60 (starting sera dilution) ID_50_ titer was reported for serum samples with no LCMV neutralization antibodies. 

### 2.7. PICV-Neutralizing Antibodies

In a black 96-well flat-bottom plate, 10,000 cells/well BHK-21 cells were seeded overnight in 100 µL of RPMI medium supplemented with 10% FBS + 1% Pen–Strep (RPMI medium) at 37 °C. Heat-inactivated sera from NHPs were diluted, and seven dilutions (four-fold), starting at 1:40 dilution, in RPMI medium were added for each sample in a 96-well U-bottom plate (sample plate). In a new plate, 3 × 10^3^ RCV of artPICV-NanoLuc virus/well was added and supplemented with diluted serum from the sample plate. Plates were incubated for 2 h at 37 °C, followed by the transfer of the virus + sample mix to previously seeded BHK-21 cells at 100 µL/well. After overnight incubation at 37 °C, the medium was aspirated, and 50 µL OPTI-MEM was added to each well.

Next, 12.5 µL per well of the diluted assay substrate (NanoGlo, Promega, Madison, WI, USA) was added to the wells, followed by luciferase acquisition on an Envision plate reader. Data were analyzed using GraphPad Prism 8.1.2, as previously described [19].

### 2.8. Detection of LCMV and PICV in Urine and Plasma

The number of RNA copies per mL was determined using the TAQMAN assay. The assay utilizes primers and a probe specifically designed to amplify and bind to conserved regions of NP and the L gene of LCMV and of PICV. The signal is compared with a known standard curve and calculated to give copies per mL, depending on the source material (i.e., plasma, urine). A 0.2 mL volume of sample (i.e., plasma, urine) was added to 0.2 mL of AL buffer with carrier RNA. A 25 µL volume of protease was added and then incubated at 56 °C degrees for 15 min. The sample was centrifuged at 11,000× *g* for 1 min and washed with wash buffer 1. The sample again was centrifuged, washed with wash buffer 2, centrifuged again, and washed with absolute ethanol. The sample was centrifuged at 17,000× *g* for 3 min to remove all alcohol. Then, 50 µL of AVE buffer was added, and the sample was centrifuged at 17,000× *g* for 1 min. The sequences for the primers and probes used to detect LCMV and PICV using the assay are described (Table 1). 

For RNA controls, the number of copies was known, so the control was diluted accordingly. Then, 20 µL of the master mix containing RNAase inhibitor and Taq-polymerase (Bioline RT-PCR kit, Taunton, MA, USA) was added with 5 µL of RNA sample to each well in a 96-well plate. The plate was sealed, and samples were tested in triplicate. 

Two curves were run with eight 10-fold serially diluted RNA controls to obtain a standard curve ranging from 1 to 10^7^ copies/reaction. 

Applied Biosystems 7500 sequence detector was used to run the reaction at a program, 48 °C for 30 min and 95 °C for 10 min, followed by 40 cycles of 95 °C for 15 s and 1 min at 56 °C. Standard curve was used to extrapolate and to calculate RNA copies per mL. Values obtained were multiplied by the reciprocal of 0.2 mL extraction volume, i.e., 50. This gave a practical range from 50 to 5 × 10^8^ RNA copies per mL. The 7500 sequence detector was calibrated at least annually by Applied Biosystems. Known standard curve was used to compare the signal and to calculate copies/mL in the samples (i.e., plasma and urine), as per the manufacturer’s recommendation.

### 2.9. Statistical Analysis

Statistical analysis was performed using GraphPad Prism 8.1.2. Non-parametric Mann–Whitney and two-way ANOVA with Šidák correction for multiple-comparison test were used for statistical analysis. We also used the Rstudio platform and lmer and emmeans packages to perform linear mixed-effects model for our analysis of longitudinal data. Statistical tests used for each dataset are indicated in the figure legends.

### 2.10. Data Availability

Data are contained within the article or Supplementary Materials.

## 3. Results

### 3.1. Heterologous Immunization with LCMV and PICV Arenavirus Vectors Induces Higher Antigen-Specific Immune Responses Compared with Homologous Administration

#### 3.1.1. SIV-Specific IFN-γ Response

**Figure 2:**
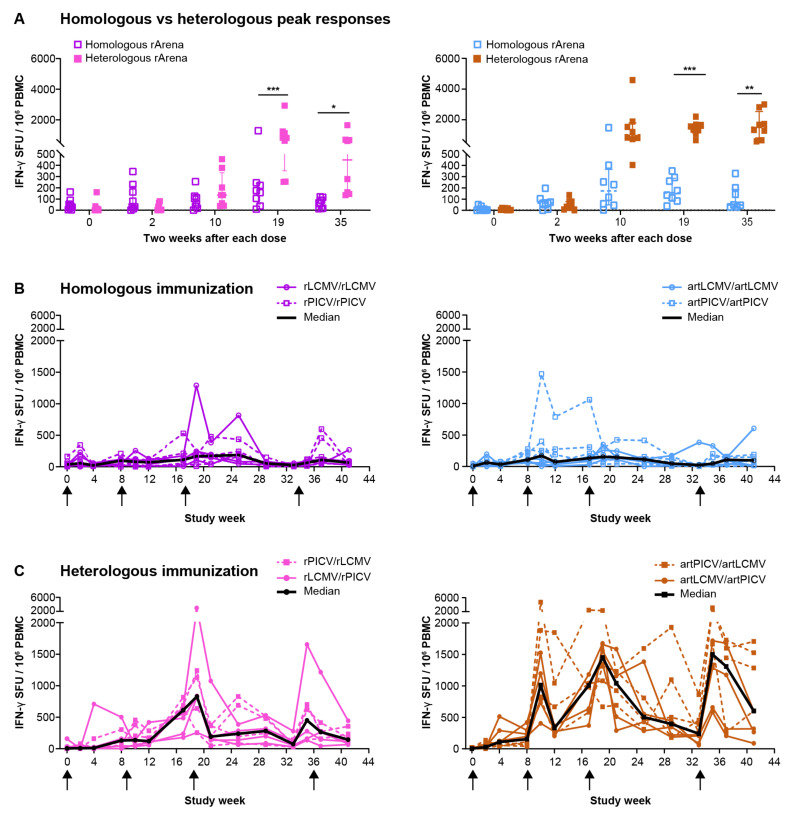
SIV-specific IFN-γ response after immunization with homologous and heterologous vaccination with replicating (art) and non-replicating (r) arenavirus vector (IV). (**A**) Total magnitude of SIV-specific (Gag + Env) IFN-γ ELISpot response at baseline and 2 weeks after each vaccine dose of homologous and heterologous immunization of rArena (upper) and artArena (lower) vector platforms. (**B**,**C**) Magnitude of SIV-specific IFN-γ response in individual NHPs after IV immunization with homologous (**B**) and heterologous (**C**) immunization of rArena (upper) and artArena (lower) arenavirus vector platforms. (**B**) Solid lines represent homologous immunization with LCMV vector, and dotted lines represent homologous immunization with PICV vector for rArena and artArena vector. (**C**) Solid lines represent heterologous immunization involving prime with LCMV vector and boost with PICV vector. Dotted lines represent heterologous immunization involving PICV prime, followed by LCMV boost. Black solid line in each graph shows median response of all animals (*n* = 4 + 4). IFN-γ ELISpot response was defined as >3× background signal; lines and error bars are median ± IQR. ELISpot responses up to week 10 were measured on frozen PBMCs, and after 10 weeks, on freshly isolated PBMCs. Statistical analysis was performed with GraphPad Prism, Wilcox test with Benjamini–Hochberg multiple correction method for panel A; * *p* = 0.02, ** *p* = 0.005 and *p* = 0.002, and *** *p* = 0.0007 and 0.0008. Heterologous LCMV and PICV vectors for prime vs. boost yielded comparable magnitude ELISpot responses via IV route; thus, both rLCMV/rPICV and rPICV/rLCMV and both artLCMV/artPICV and artPICV/artLCMV group responses were combined into a single group (each *n* = 8).

We initiated studies to determine the immunogenicity of arenavirus vectors encoding SIV_smE543_ Gag and Env immunogens in rhesus macaques. To evaluate whether repeated homologous or heterologous immunization with either artLCMV/artPICV) or rLCMV/rPICV would generate cellular responses to SIV_smE543_ Gag and Env immunogens in naïve NHPs, we performed IFN-γ ELISpot assays 2 weeks after each IV immunization. For both vector platforms administered IV, heterologous immunization with LCMV and PICV vectors led to a significantly higher total (Gag + Env) magnitude of IFN-γ ELISpot response in PBMC than homologous immunization (artArena, *p* < 0.0001, rArena, *p* = 0.02, Tukey method, and linear mixed-effects model for curve comparison; Supplementary Figure S1). The responses were significant after the third vaccination dose (at week 19 and 35) for both vector platforms (Figure 2A). These data suggest that heterologous immunization of both vector platforms elicits SIV-specific cellular responses more efficiently than homologous immunization. Initiating the heterologous regimen with either LCMV or PICV yielded a comparable magnitude of IFN-γ ELISpot responses by the IV route (Figure 2C and Supplementary Figure S3); thus, rLCMV/rPICV and rPICV/rLCMV and both artLCMV/artPICV and artPICV/artLCMV group responses were combined into a single group (each *n* = 8) for further comparisons to increase statistical power for T-cell ELISpot analysis, and they are referred to as rPICV/rLCMV and artPICV/artLCMV, respectively, throughout the manuscript. Similarly, the homologous immunization with LCMV and PICV for either of the vector platforms yielded a comparable magnitude of IFN-γ response (Figure 2B and Supplementary Figure S2); hence, they were combined for statistical analysis (Figure 2A).

#### 3.1.2. Vector Viral Load, Persistence, and Shedding

**Figure 3:**
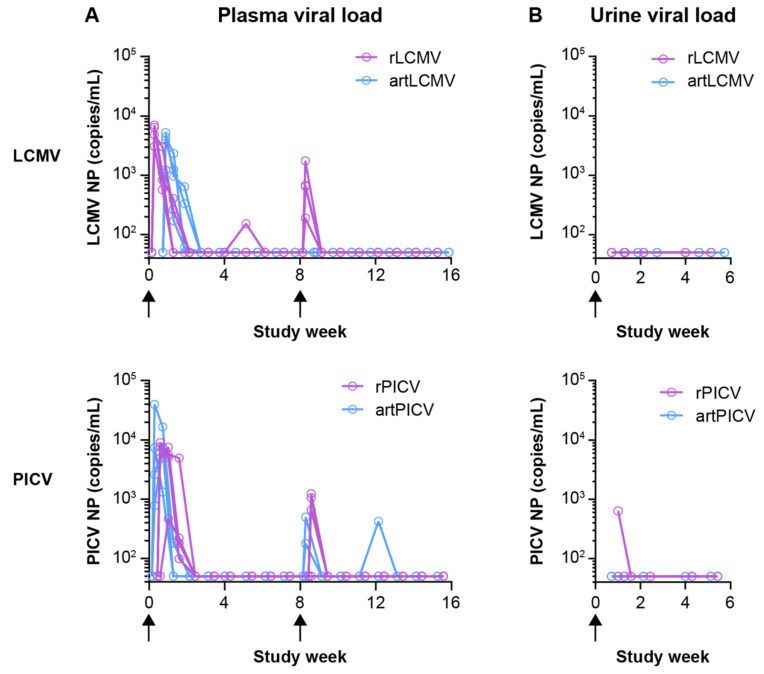
LCMV and PICV vector kinetics in blood and urine (IV). Four macaques per group were immunized IV with rLCMV, rPICV, artLCMV, and artPICV vectors encoding SIV_smE543_Gag and SIV_sme543_Env in a homologous regimen on study weeks 0 and 8. At the indicated timepoints, samples were collected, and viral NP gene copies measured by qRT-PCR. Individual animal NP copies per mL of plasma (**A**) and urine (**B**) per group are plotted. Arrows indicate vaccine-dose timepoints.

To determine persistence of arenavirus vectors in NHPs, we evaluated the kinetics of detectable viral vectors in the plasma and their shedding into urine for both rArena and artArena vectors after homologous IV immunization. Viremia was detectable in the plasma of all animals and was cleared by 6 weeks after the initial dose, as determined by LCMV- or PICV-NP gene copies measured by qRT-PCR. Viral vector genomes were only detectable in the urine of one animal at one timepoint (1 week) after dosing with rPICV and became undetectable by 2 weeks or sooner (Figure 3B). In summary, vector genomes were detectable in plasma (for all the vectors tested) and urine (for rPICV), peaking within the first 1–2 weeks after dosing and declining to undetectable levels by, at most, 6 weeks after the first two doses of vaccination. 

### 3.2. artArena Immunization Induces Higher SIV-Specific Responses Than rArena Vectors

#### 3.2.1. SIV-Specific IFNγ ELISpot Response

**Figure 4:**
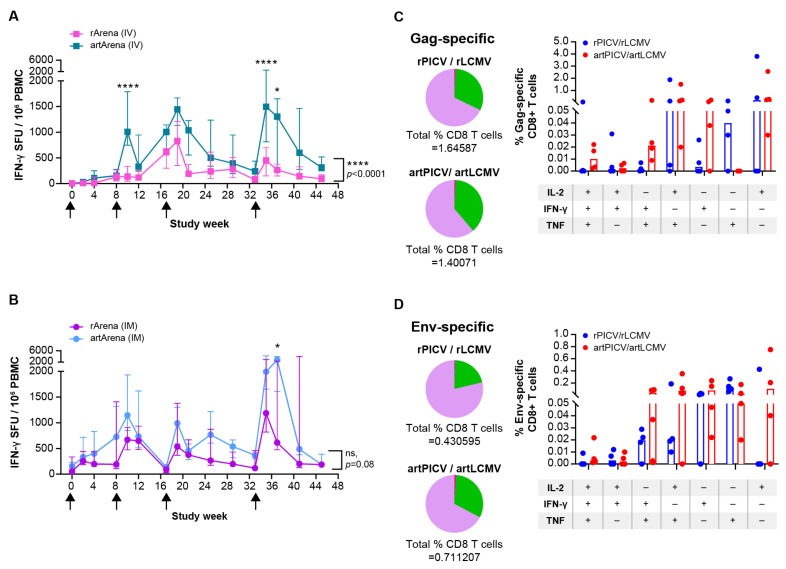
Comparison between SIV immunogenicity elicited after immunization with artArena and rArena arenavirus vector administered IV (**A**) and IM (**B**). Heterologous LCMV and PICV vectors for prime vs. boost yielded comparable magnitude ELISpot responses by IV route; thus, both the rLCMV/rPICV and rPICV/rLCMV and both artLCMV/artPICV and artPICV/artLCMV group responses were combined into a single group (each *n* = 8) and referred as rPICV/rLCMV and artPICV/artLCMV, respectively. ELISpot responses up to week 10 were measured on frozen PBMCs, and after 10 weeks, on freshly isolated PBMCs for IV groups. Freshly isolated PBMCs were evaluated for IM groups. Black arrows represent PICV vector immunization. Statistical significance for each timepoint was determined by two-way ANOVA Šidák multiple-comparison test, and for curve comparison, linear mixed-effect model was used. **** *p* < 0.0001, * *p* < 0.05 (**A**) Median responses of all the animals of heterologous IV group of both artArena and rArena vector groups, side by side, on the same graph from Figure 2C for comparison and statistical analysis. (**C**,**D**) SIV-specific Gag (**C**) and Env (**D**) CD8 T-cell polyfunctionality after fourth vaccination of rPICV/rLCMV and artPICV/rLCMV vector (IV) in NHPs (*n* = 4 each group). Intracellular cytokines evaluated include IFN-γ, IL2, and TNF-α in CD8 T cells by multiparametric flow cytometric analysis. Frequencies of T cells positive for single (purple color), double (green color), and triple (pink color) cytokines are plotted as pie charts.

Next, we investigated differences between the two vector platforms for generating cellular responses to SIV_smE543_ immunogens when administered as heterologous vector therapy. The IV administration of artArena vectors resulted in higher total SIV-specific (Gag + Env) IFN-γ ELISpot responses than rArena vector therapy (*p* < 0.0001, linear mixed model for curve comparison). These differences were most significant after the second and fourth doses (*p* < 0.0001, two-way ANOVA, Šidák multiple comparison; Figure 4A). When compared separately for both Gag and Env antigens, artPICV/artLCMV elicited a higher magnitude of IFN-γ T-cell response to both the antigens compared with rPICV/rLCMV vectors (Supplementary Figure S4).

We also investigated the effect of the vaccination route on SIV-specific cellular responses for comparing both vector platforms. To this end, we analyzed the magnitude of total (Gag + Env) IFN-γ ELISpot responses elicited by artArena and rArena vectors when administered IM. The artPICV/artLCMV vectors consistently induced higher cellular responses than rPICV/rLCMV, but these differences became statistically significant only after the fourth dose (*p* < 0.05, two-way ANOVA Šidák multiple-comparison test; Figure 4B and Supplementary Figure S5).

#### 3.2.2. SIV Env-Specific Binding IgG Response

To investigate differences between artArena and rArena vectors in eliciting anti-SIV antibody response, we determined IgG titers of autologous (SIV_smE543_) and heterologous (SIV _smE660_ and SIV_mac251_) anti-SIV Env-binding antibodies in the sera of NHPs after each heterologous immunization. We observed a significant difference in anti-SIV antibody induction between artArena and rArena vaccination when the vectors were administered IV, with artArena vectors being the more potent inducers (Supplementary Figure S6A; SIV_smE543_ *p* = 0.016, SIV_mac251_ *p* = 0.052, and SIV_smE660_ *p* = 0.033, linear mixed model for curve comparison). Interestingly, these differences were not significant when the same immunization was administered IM (Supplementary Figure S6B). 

#### 3.2.3. SIV-Specific Monofunctional and Polyfunctional T-Cell Responses

To evaluate the ability of the arenavirus vectors to elicit a functional T-cell response, we determined Gag- and Env-specific monofunctional (one cytokine) and polyfunctional (two or three cytokines) CD8 and CD4 T-cell responses 2 weeks after the fourth vaccination dose in the heterologous regimen groups, rPICV/rLCMV and artPICV/artLCMV (IV). The rPICV/rLCMV and artPICV/artLCMV vectors showed similar levels of SIV-specific IFN-γ, IL-2, or TNF-α production in CD8 T cells (Supplementary Figure S7), in addition to polyfunctional CD8 T-cell responses. While the differences were not significant given the small cohort size (*n* = 4), the artPICV/artLCMV vectors showed slightly higher percentages of CD8 T cells for each cytokine evaluated (Figure 4C,D). SIV Gag- and Env-specific monofunctional and polyfunctional CD4 T-cell responses were lower compared with CD8 T-cell responses (Supplementary Figure S8) for the same antigen, suggesting arenavirus vector favor CD8 T-cell responses. The rArena vectors elicited a slightly higher Gag-specific overall (non-significant) CD8 T-cell response than the artArena vectors. However, most of those CD8 T cells exhibited single-cytokine responses. In comparison, the proportion of Gag-specific CD8 T cells exhibiting two-cytokine and three-cytokine responses (polyfunctional response) was larger upon vaccination with artArena vectors.

### 3.3. IM Administration of Arenavirus Vectors Elicits a Robust CD8 T-Cell Response with Breadth against SIV Gag and Env Antigens

**Figure 5:**
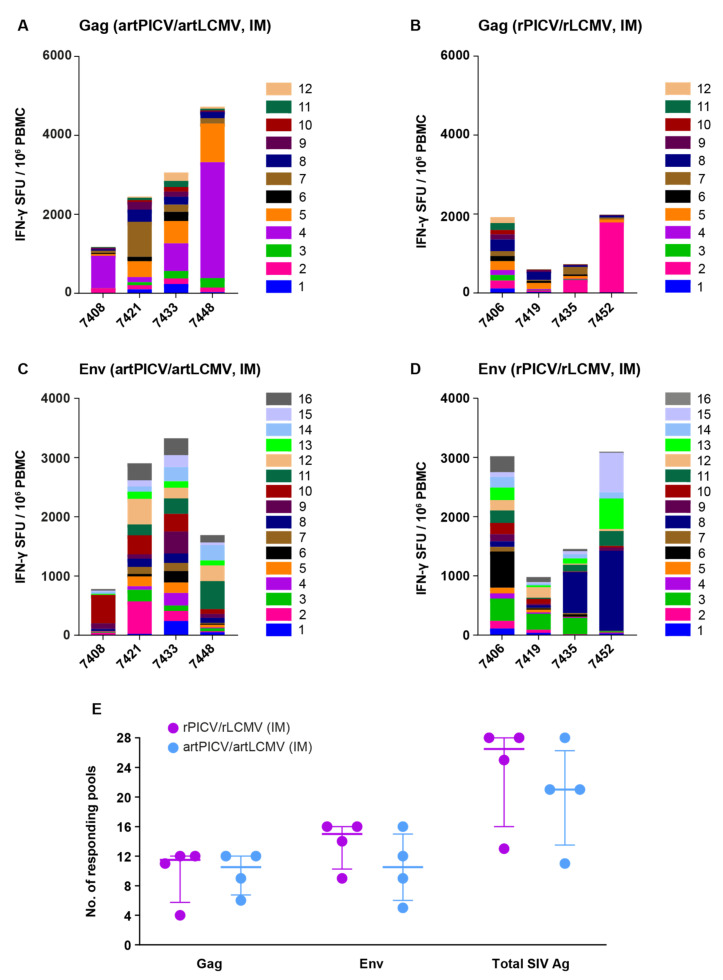
Breadth of SIV-specific responses 2 weeks after the fourth dose of heterologous immunization of rPICV/rLCMV and artPICV/artLCMV vectors when administered IM. ELISpot response breadth was evaluated using 12 Gag (**A**,**B**) and 16 Env peptide subpools (**C**,**D**) at 2 weeks after fourth dose of rPICV/rLCMV and artPICV/artLCMV IM; response was defined as >3× background signal; lines and error bars represent the median ± IQR. (**E**) Responses to total number of Gag and Env pools (10 MER pools) elicited after IM immunization with SIV immunogen encoding rPICV/rLCMV and artPICV/artLCMV vectors.

The breadth of vaccine-induced cellular immunity has been shown to correlate with better efficacy against SIV in NHP studies using adenoviral vector SIV vaccine (Ad26) [6]. To characterize T-cell breadth upon arenavirus vaccination, we determined the cellular immune responses to 12 SIV-specific Gag and 16 Env peptide subpools matched with the vaccine immunogen. Both vector platforms induced IFN-γ ELISpot responses against 9 to 28 of 28 (sum of Gag and Env peptide pools) possible antigen subpools measured at 2 weeks after the fourth vaccination dose (Figure 5). Overall, our data demonstrate that arenavirus vectors elicit broad responses to lentiviral immunogens, with no statistical differences observed between the two platforms. 

### 3.4. IM Heterologous SIV Immunization of artPICV/artLCMV Arenavirus Vectors Generates Overall Superior Vaccine Responses Compared with IV Immunization

#### 3.4.1. Humoral Responses to Immunogen and Vector Platform

**Figure 6:**
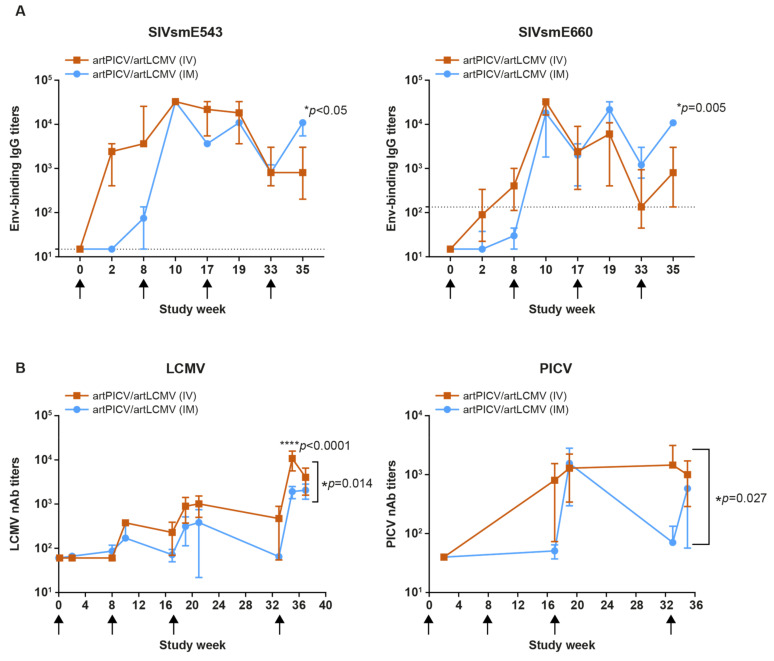
Comparison between heterologous immunization of artPICV/artLCMV and rPICV/rLCMV vector for generating SIV envelope binding IgG titers and anti-vector (LCMV and PICV) neutralization antibody titers. Binding anti-Env IgG titers against autologous SMe543 and heterologous SIVsmE660 gp120 after IV and IM immunization (**A**). Anti-LCMV and anti-PICV vector neutralization Ab titers (**B**). Data represent anti-Env immunoglobulin-G (IgG) end-point titers over the course of study plotted as median ± IQR (*n* = 4). Statistical significance for each timepoint was determined by two-way ANOVA Šidák multiple-comparison test, and for curve comparison, linear mixed-effect model was used. * and **** *p* values are mentioned on the graph for significance. Black arrows represent PICV vector immunization.

Given the advantage that we observed for artPICV/artLCMV vectors over rPICV/rLCMV vectors in generating vaccine-specific T-cell responses, we next determined whether the route of administration affects SIV vaccine response. First, we investigated the potential of artPICV/artLCMV arenavirus vectors when given IV or IM to elicit SIV-specific antibodies by determining the end-point titers of autologous (SIV_smE543_) and heterologous (SIV_smE660_ and SIV_mac251_) Env-binding antibodies in the serum of NHPs after SIV immunization using a heterologous regimen. Both IV and IM immunization led to generation of strong autologous and heterologous Env-binding IgG titers (Figure 6A and Supplementary Figure S6). IM immunization resulted in antibody responses that were delayed but also more stable relative to IV immunization. Antibody titers were also augmented and maintained with IM immunization compared with IV after the fourth vaccination dose. A significant difference in titers between IV and IM administration was observed at 2 weeks after last immunization for both homologous (SIV_smE543_) and heterologous (SIV_smE660_) SIV Env antigens with heterologous immunization of artPICV/artLCMV vectors (Figure 6A).

Second, we evaluated the induction of anti-LCMV and anti-PICV neutralizing antibodies in NHPs after heterologous IV or IM immunization with artLCMV and artPICV. Of note, heterologous IM immunization with artLCMV and artPICV arenavirus vectors resulted in significantly lower (*p* < 0.0001, linear mixed model for curve comparison) anti-LCMV neutralizing antibodies (nAbs) than IV immunization (Figure 6B). Anti-PICV nAb induction was similarly lower when artArena vectors were administered IM (vs. IV), as measured at weeks 17 and 33 (Figure 6B). Also, IV immunization maintained higher titers of anti-PICV neutralizing antibodies over the course of the timepoints evaluated when compared with IM immunization (*p* = 0.027, linear mixed model for curve comparison), after which the titers decreased much more rapidly by 4 weeks after the last vaccination (Figure 6B). 

To determine if anti-vector nAbs affected SIV antigen-specific immune responses, we evaluated a potential correlation of anti-vector nAb titers with SIV IFN-γ ELISpot at 2 weeks after last vaccination (Supplementary Figure S9). Interestingly, increased LCMV nAb titers showed no significant correlation with SIV antigen-specific T-cell responses. 

#### 3.4.2. SIV-Specific and Anti-Vector Cellular Immunogenicity

**Figure 7:**
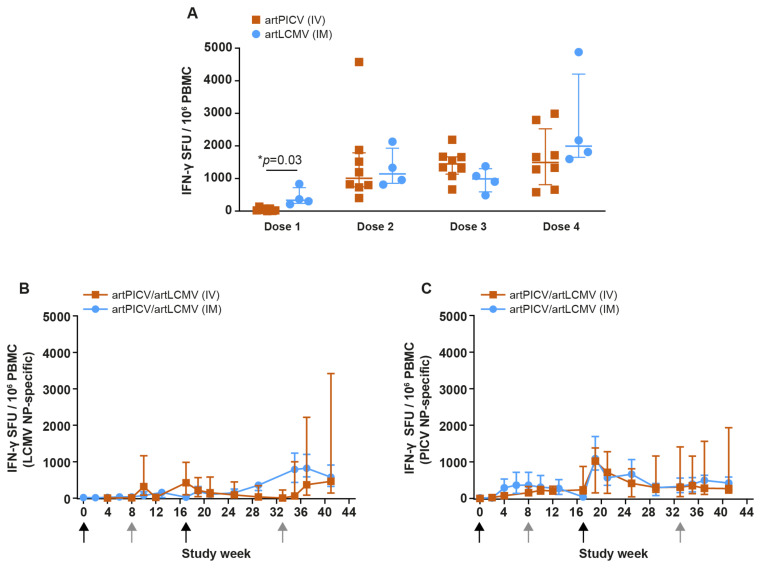
Comparison between IV and IM immunization of artPICV/artLCMV vectors for generating SIV-specific IFN-γ ELISpot in naïve NHPs. (**A**) SIV-specific IFN-γ responses 2 weeks after each dose of IV and IM heterologous immunization of replicating vector. (**B**) LCMV NP-specific IFN-γ ELISpot response. (**C**) PICV NP-specific IFN-γ ELISpot response after immunization with heterologous boost of artPICV/artLCMV arenavirus IV and IM. Data represent magnitude of anti-vector NP-specific IFN-γ ELISpot response (**B**,**C**) over the course of study, plotted as median ± IQR (*n* = 4). Statistical significance for timepoint was determined by Wilcox with Benjamini–Hochberg multiple correction test * *p* = 0.03. Black arrows represent PICV vector immunization, and grey arrows represent LCMV vector immunization.

Next, we compared the magnitude of SIV-specific IFN-γ ELISpot after heterologous IV and IM immunization with artPICV/artLCMV arenavirus vectors. Both the IV and IM administration resulted in a continuous increase in SIV-IFN-γ response after each vaccination dose. A trend for a stronger increase (not statistically significant) in the overall magnitude of total SIV-specific IFN-γ response was observed with IM immunization compared with IV immunization (after fourth vaccination dose; Figure 7A).

Additionally, we determined vector-specific (LCMV-NP and PICV-NP) IFN-γ ELISpot responses after each immunization. Moderate vector-specific T-cell responses were detected after heterologous artArena IM or IV immunization (Figure 7B,C), with no significant difference between the IM and IV route of administration. Of note, when comparing total SIV-specific T-cell responses (against Gag and Env) and vector backbone-specific responses, backbone T-cell responses appeared at a significantly lower magnitude (two-way ANOVA, Dunnett multiple-comparison test, *p* < 0.0001; Supplementary Figure S10).

## 4. Discussion

In this study, we demonstrate that arenavirus vaccine vectors encoding SIV_smE543_ Gag and Env effectively induce cellular and humoral immunity in treatment-naïve rhesus macaques. To determine the optimal vaccination setting for upcoming studies and potential future clinical development, we investigated and compared the potency of artArena and rArena vectors to elicit SIV-specific immune responses in heterologous and homologous regimens and with two different routes of administration (i.e., IV and IM). Heterologous (alternating PICV/LCMV) immunization with artArena or rArena vectors stimulated significantly stronger T-cell responses when compared with homologous administration (PICV/PICV or LCMV/LCMV), as evident from comparison of magnitude of IFN-γ ELISpot responses against both encoded SIV antigens (Env and Gag; Figure 2 and Supplementary Figure S1). To determine a limited safety profile for artArena and rArena vectors after IV administration, we measured viremia and virus shedding (in urine) in these animals by RT-qPCR and detection of viral genomes at various timepoints. Vectors are rapidly cleared from blood (Figure 3A) for both artPICV/artLCMV and rPICV/rLCMV vaccines. Furthermore, viral shedding into urine was detected only for one animal at a single timepoint (Figure 3B) after rPICV administration. Body weight and temperature were also monitored for all the animals throughout the study, and no abnormalities were observed. Additionally, a previous clinical study found non-replicating arenavirus vectors to be safe and well tolerated [17], which aligns with our observations in the present study, as well as other NHP studies with replicating arenavirus vectors [19]. These data support the strong attenuation and good safety profile of both arenavirus vector platforms. 

We further compared two different routes of administration (IM and IV) and discovered that repeated IM administration of PICV and LCMV vectors resulted in a more stable antigen (SIV Env)-specific antibody response and lower induction of anti-vector antibodies than repeated IV administration of the vaccine. When administered intravenously, artArena vectors induced significantly higher SIV antigen-specific T-cell and antibody responses than rArena vectors. Interestingly this difference (between rArena and artArena) was much less pronounced in monkeys that received IM dosing (Figure 4A,B). The different kinetics of IgG and T-cell responses after IM versus IV dosing are an interesting and unexpected observation. It has been shown that arenaviruses preferentially infect APCs among PBMCs [22]. Whether IM dosing leads to different target cells compared with IV dosing and delayed transport of the antigen to APCs in draining LNPs is currently unknown and the subject of ongoing preclinical research. It also should be noted that the observed differences in the T-cell response could be due to fresh/frozen samples. 

Of note, the quality of a T-cell response, as defined by the polyfunctionality rather than the quantity of T cells, has been associated with effective immune responses against viral infections and disease progression [23]. And interestingly, polyfunctional CD8+ T cells have been shown to be associated with better viral control in elite HIV controllers and may be critical for therapeutic HIV vaccine efficacy [23,24]. Those functional effector T cells produce cytokines such as IFN-γ, IL-2, MIP-1β TNF-α, and CD107a, which, in combination, aid in viral inhibition and potentiate the immune response by activating other immune cells.

Here, we demonstrate that both artArena- and rArena-based heterologous immunization regimens induced SIV-specific polyfunctional CD8 T-cell responses. The strong induction of polyfunctional T-cell responses after heterologous immunization with artArena vectors was recently confirmed in a larger NHP cohort (*n* = 24) SIV efficacy study published by our group. This study links the potent induction of cellular (polyfunctional T cells and T-cell breadth) and humoral (antibody) responses, induced by artLCMV and artPICV vectors encoding SIV antigens, to a significant reduction in SIV viral load for peak and setpoint in SIV_mac251_-challenged animals [19]. In the present study, we recapitulated the magnitude and breadth of the SIV-specific T-cell response against the encoded immunogens and investigated different regimens and routes of administration with both artPICV/artLCMV and rPICV/rLCMV vectors to determine the optimal vaccination setting. We found heterologous immunization with artArena vectors (alternating between PICV and LCMV) to be superior in regard to inducing T-cell responses when compared with rArena heterologous immunization. Interestingly, this difference was more pronounced after IV administration (Figure 4A,C,D). This could be due to the slightly larger cohort size (*n* = 8) for IV immunization that was achieved by combining the groups compared with IM immunization (*n* = 4). 

Generally, we observed higher SIV CD8 T-cell response than CD4 T-cell in our study, which further illustrates the potential of arenavirus vectors in generating strong CD8 T-cell immunity, as reported previously in cancer models in mice [20] and in clinical-trial settings [18].

Viral vectors such as Ad5 have been shown to produce vector-neutralizing antibodies that limit the effectiveness of T-cell response in individuals with pre-existing immunity to the vectors or prevent repeated administration with same vectors [25,26]. In our study cohorts, we measured anti-LCMV and anti-PICV neutralizing antibodies after immunization with artPICV/artLCMV arenavirus vectors and their impact on vaccine-induced T-cell responses. Although we observed anti-vector-neutralizing antibodies with repeated dosing, they did not negatively impact SIV-specific T-cell responses (Supplementary Figure S9), as we observed that boosting with LCMV- or PICV-based vectors further enhanced cellular immune responses (Figure 2). This indicates that the first exposure to these vectors did not prevent them from boosting the response to the immunogen and suggests that anti-vector antibodies against these arenavirus vectors do not affect vaccine antigen responses when delivered as a heterologous prime–boost regimen. Additionally, a larger cohort of 24 NHPs was tested in the scope of the aforementioned efficacy study [19], and similarly, repeated, alternating administration of artPICV and artLCMV vectors induced anti-LCMV neutralization antibodies but did not negatively impact SIV immunogenicity, as shown by an increase in SIV immunogen-specific humoral and cellular responses. A potential explanation for our observation is the internalization of viral vector–immune complexes (comprising vector and anti-vector nAbs) via FcgR into APCs. This uptake could account for the generation of vaccine-specific responses in the presence of a low level of anti-vector nAbs, as has been hypothesized previously [27]. A similar mechanism leading to SIV-specific T-cell and B-cell response in the presence of vector nAbs might be responsible for the lack of correlation between SIV T-cell response and vector nAbs in the present study. 

Interestingly, in a phase 1 clinical trial of a rLCMV vector-based vaccine against human cytomegalovirus, no LCMV-neutralizing antibodies were detected after repeated homologous dosing of the vaccine [17]. These differences in observation likely originate from (i) virological and biological differences between artArena and rArena vectors and (ii) differences in species. It was reported that subclinical LCMV infection in rhesus macaques stimulated adaptive immunity that included virus-binding antibodies and cell-mediated immunity [28]. Concurrent with LCMV and PICV nAbs, we also detected vector backbone-specific T-cell responses, as measured by IFN-γ ELISPOT response against LCMV and PICV NP (Figure 7B,C). While the route of administration had no detectable impact on the induction of vector NP-specific T-cell responses, we observed significantly lower vector nAb titers upon IM administration (Figure 6B). An impact of vector-specific cellular and humoral immunity on antigen-specific responses cannot be ruled out, as the repeated administration of the same vector (homologous regimen) results in significantly lower SIV-specific T-cell responses compared with heterologous administration of the two phylogenetically distant vectors (artPICV and artLCMV), directing the cellular response toward the disease-specific antigens and away from responses to the backbone (Supplementary Figures S10 and S11). This confirms previous findings in mouse tumor models [20].

This suggests that the arenavirus vector platform can provide an advantage over other viral vectors, for which repeat dosing poses an issue due to pre-existing immunity against the vector platform. However, more research is needed to determine (i) the full extent of the advantages of the arenavirus platform over other platforms for vaccine development. Although an SIV virus challenge was not conducted in this study, a strong induction of polyfunctional T-cell responses after heterologous immunization with artArena vectors was recently confirmed in a larger NHP cohort (*n* = 24) SIV efficacy study published by our group. This study links the potent induction of cellular (polyfunctional T cells and T-cell breadth) and humoral (antibody) responses induced by artLCMV and artPICV vectors encoding SIV antigens to a significant reduction in SIV viral load for peak and setpoint in SIVmac251-challenged animals [19]. Based on the preclinical safety, immunogenicity, and efficacy profile of the vaccine, a phase 1b clinical trial employing artPICV/artLCMV arenavirus vectors that encode conserved regions of HIV antigens is planned (NCT06430905).

The nature of arenaviruses (e.g., LCMV and PICV) to infect professional APCs, such as dendritic cells and macrophages [8,10], supports several strategies that could be explored to further potentiate the immunogenicity of arenaviral vectors. For example, immune modulators that further activate, expand, or shape dendritic cells, macrophages, or T cells and their interaction could potentially increase the vaccine immunogenicity of such vectors. Furthermore, arenaviruses could potentially be combined with complementary viral vectors, as suggested by a study that used a recombinant Ad5 vector prime followed by an rLCMV boost [29]. These findings suggest that SIV vaccine immunogenicity can be further enhanced by combining arenavirus vectors with other vaccine platforms, like chimpanzee adenoviral (ChAd) vectors, modified vaccinia vectors, or mRNA-based lipid nanoparticles.

## 5. Conclusions

Overall, our study demonstrated that repeated dosing with heterologous LCMV- and PICV-based vectors generates strong SIV-specific T-cell responses compared with a homologous regimen. Anti-vector immunity was observed but was lower after IM administration, and it did not diminish vaccine responses using either route. The artPICV/artLCMV vectors showed significantly better T- and B-cell response than rPICV/rLCMV vectors. The robust and broad CD8 T-cell immunity generated by these vectors, in combination with the limited effects of anti-vector immunity, indicate that they could provide a promising vaccine-delivery platform for HIV treatment. These results support the use of LCMV- and PICV-based vectors for the enhancement of HIV-specific immunity in a therapeutic setting.

## Figures and Tables

**Table 1:** Sequences for the primer/probe sets.

Primer/Probe Set	Sequence (5′ to >3′)
LCMV-NP fwd	TGCGGAAGAGCACCTATAACTG
LCMV-NP rev	TTGCCGACCTCTTCAATGC
LCMV-NP probe	CGAGGTCAACCCGG
LCMV_cl13_L_fwd	ACTGGAGTCAGATCGCTGATGAG
LCMV_cl13_L_rev	TGTTAAGTGGAAAAGGGATGAACATT
LCMV_cl13_L_probe	AGGTCAGAAAACAGAACAGT
PIC_NP fwd	GCCATTTCCACCGGATCA
PIC_NP rev	ATTCAAAAGCTACCACATGGATTG
PIC_NP probe	TTGGTGTTCCTTCAATG
PIC_L_fwd	AAGTGATTGGGATTGTTTAGGTGAGT
PIC_L_rev	TGCTTGCGAGTTGGGTAACTG
PIC_L_probe	ACTATCTTGGGTACTTCAGCT

## Data Availability

Data are contained within the article or Supplementary Materials.

## Supplementary Materials

The following supporting information can be downloaded at https://www.mdpi.com/article/10.3390/vaccines12070735/s1, Supplementary Figure S1: Median responses after vaccination with homologous and heterologous immunization of replicating and non-replicating vector platforms. Supplementary Figure S2: SIV-specific IFN-γ response after immunization with homologous vaccination with art and r arenavirus vector (IV). Supplementary Figure S3: SIV-specific IFN-γ response after immunization with heterologous vaccination with art and r arenavirus vector (IV). Supplementary Figure S4: SIV-specific IFN-γ response after immunization heterologous immunization with replicating and non-replicating arenavirus vector (IV). Supplementary Figure S5: SIV-specific IFN-γ response after immunization heterologous immunization with replicating and non-replicating arenavirus vector (IM). Supplementary Figure S6: Comparison between heterologous immunization of artPICV/artLCMV and rPICV/rLCMV vector for generating SIV envelope binding IgG titers. Supplementary Figure S7: Monofunctional CD8 responses. Supplementary Figure S8: SIV-specific (Env ang Gag) CD4 T-cell polyfunctionality after fourth vaccination of rPICV/rLCMV and artPICV/artLCMV (IV) in NHPs. Supplementary Figure S9: Anti-vector immunity. Correlation between SIV-specific IFN-γ ELISpot and LCMV vector nAbs at week 35 (2 weeks after last vaccination dose) for replicating arenavirus vectors. Supplementary Figure S10: Magnitude of SIV-specific IFN-γ responses were higher than vector-specific NP response (IV).
