# Supplementary material for: Arenavirus-Based Vectors Generate Robust SIV Immunity in Non-Human Primates
Source: Vaccines (Basel). 2024 Jul 2;12(7):735. doi: 10.3390/vaccines12070735 (PMC11281402; doi:10.3390/vaccines12070735)
Supplement: Supplementary file 1 [file vaccines-12-00735-s001.zip › vaccines-3026209-supplementary.pdf]

SUPPLEMENTARY INFORMATION

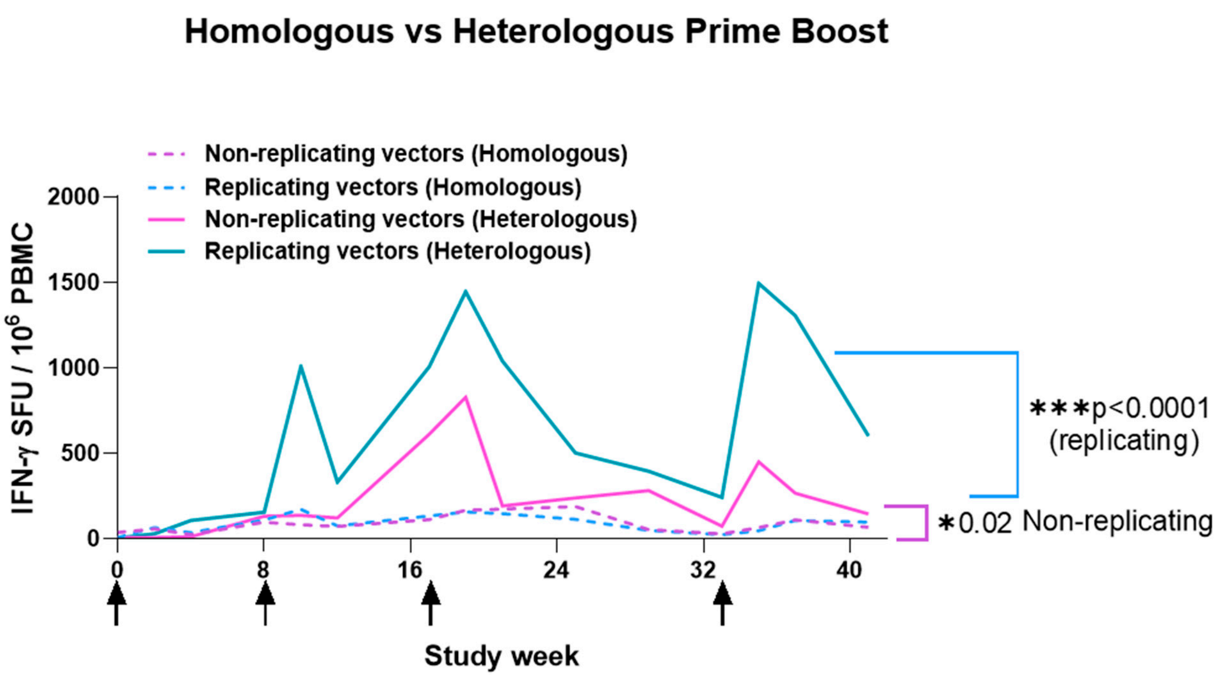

Supplementary Figure S1. Median responses after vaccination with homologous and heterologous immunization of replicating and non-replicating vector platforms. Curves were compared using linear mixed-effect model with adjusted *p* values, Tukey test. Heterologous LCMV and PICV vectors for prime vs. boost yielded comparable magnitude ELISpot responses by IV route; thus, both non-replicating (rLCMV/rPICV and rPICV/rLCMV) and both replicating (artLCMV/artPICV and artPICV/artLCMV) group responses were combined into a single group (each $n = 8$). Homologous groups for each vector type, replicating (artLCMV, 4+artPICV, 4) and non-replicating (rLCMV, 4+rPICV, 4) were combined here for median responses.

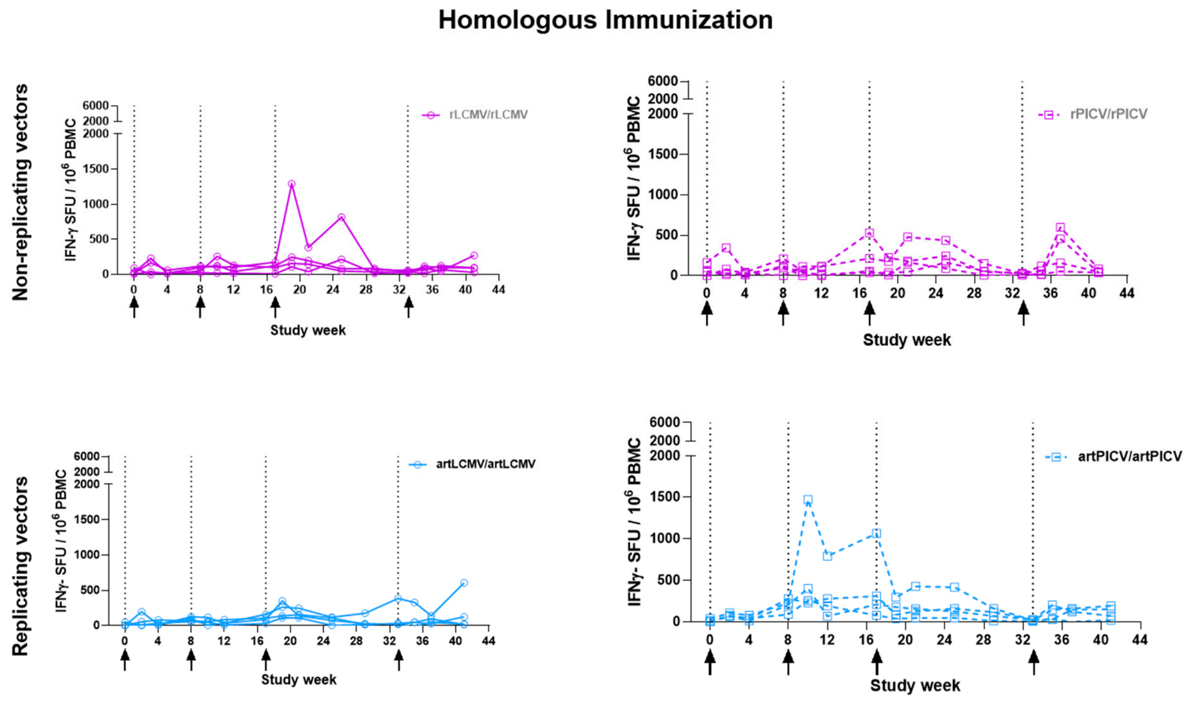

Supplementary Figure S2. SIV-specific IFN-γ response after immunization with homologous vaccination with replicating (art) and non-replicating (r) arenavirus vector (IV). Magnitude of SIV-specific IFN-γ response in individual NHPs after IV immunization with homologous immunization of non-replicating (upper panel) and replicating (lower panel) arenavirus vector platforms. Solid lines represent homologous immunization with LCMV vector and dotted lines represent homologous immunization with PICV vector for replicating (blue) and non-replicating (purple) vector.

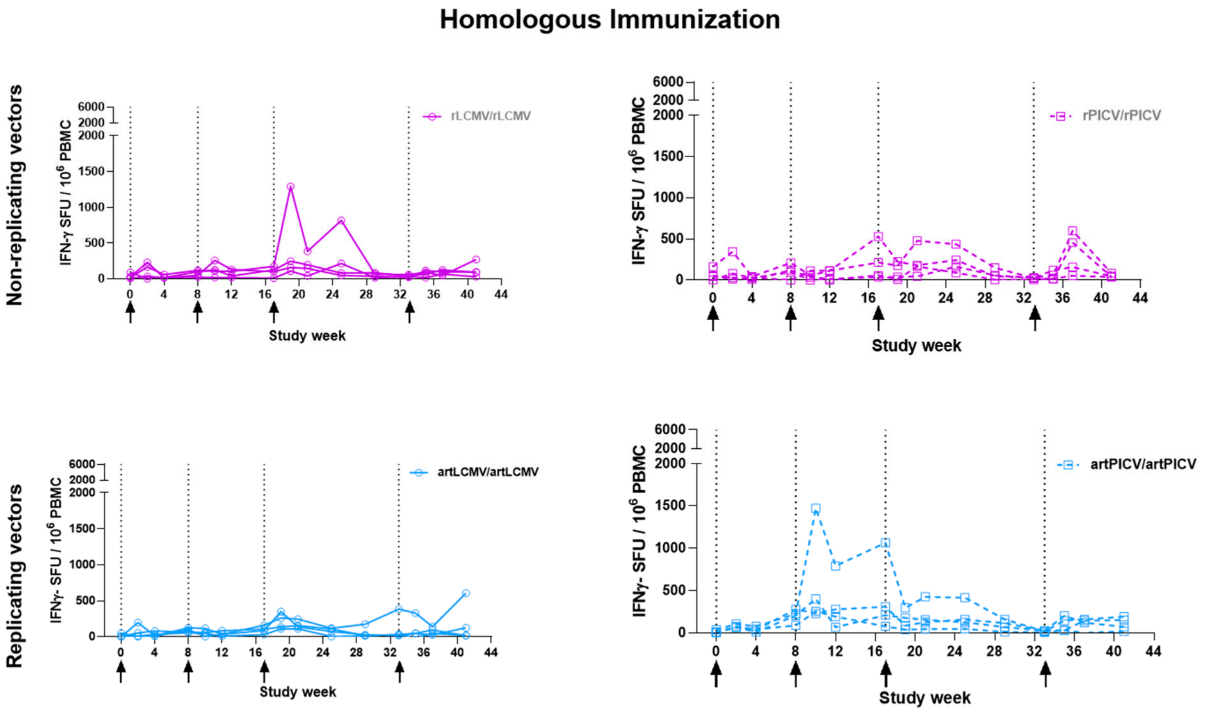

Supplementary Figure S3. SIV-specific IFN-γ response after immunization with heterologous vaccination with replicating (art) and non-replicating (r) arenavirus vector (IV). Magnitude of SIV-specific IFN-γ response in individual NHPs after IV immunization with homologous immunization of non-replicating (upper panel) and replicating (lower panel) arenavirus vector platforms. Solid lines represent heterologous immunization vector with non-replicating (pink) and replicating (cyan) involving prime with LCMV vector and boost with PICV vector. Dotted lines represent heterologous immunization involving PICV prime followed by LCMV boost.

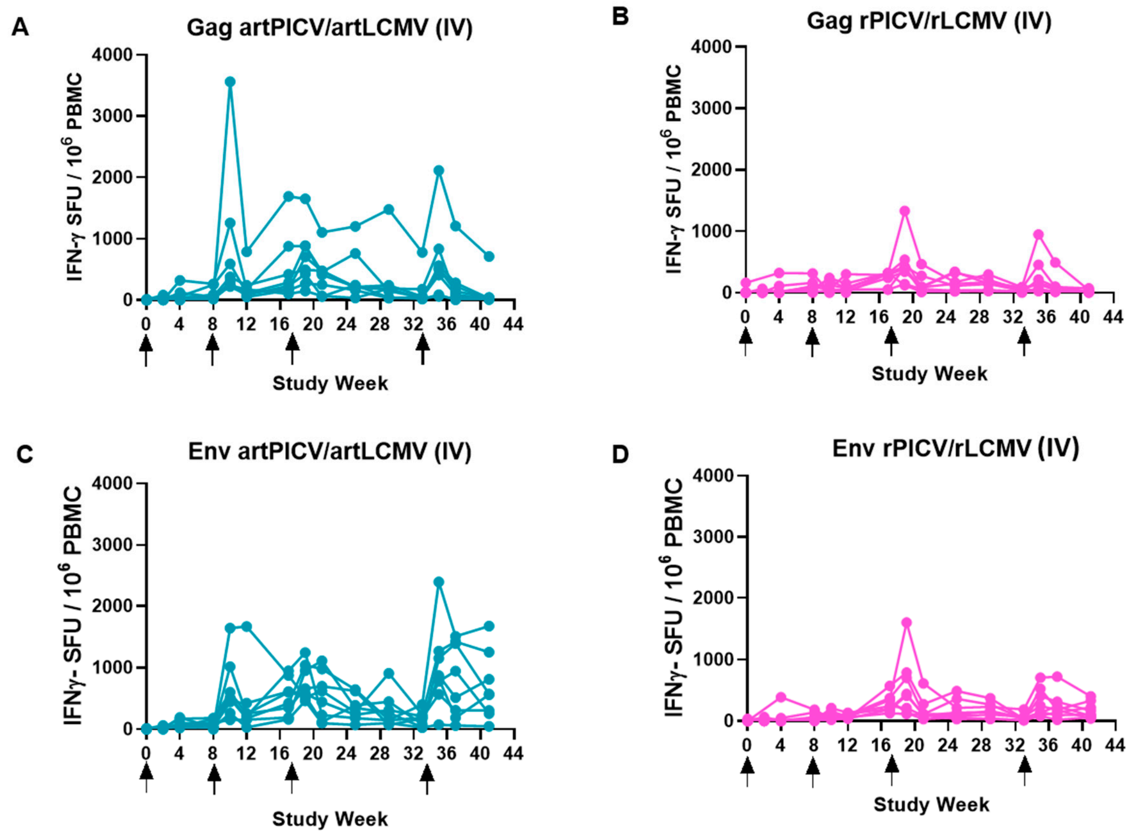

Supplementary Figure S4. SIV-specific IFN-γ response after immunization heterologous immunization with replicating and non-replicating arenavirus vector (IV). (**A** and **B**), Gag-specific IFN-γ ELISpot responses in individual animals after heterologous immunization with replicating (**A**) and non-replicating (**B**) arenavirus vector platforms. (**C** and **D**), Env-specific IFN-γ ELISpot response in individual animals after heterologous immunization with replicating (**C**) and non-replicating (**D**) arenavirus vector platforms.

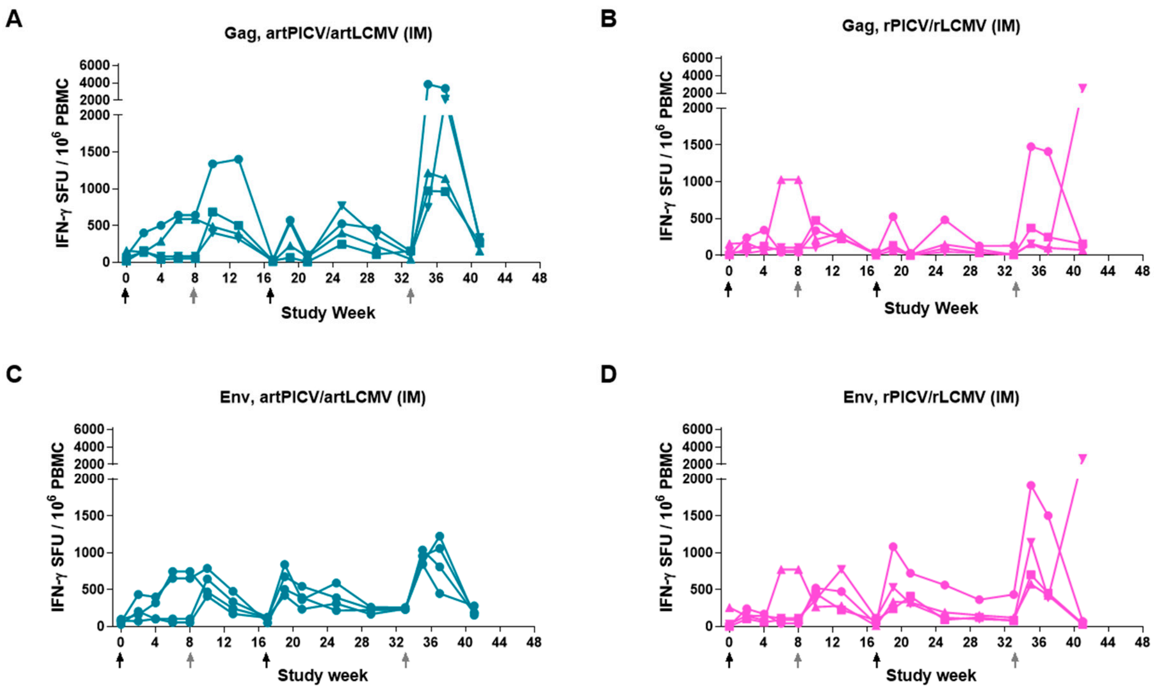

Supplementary Figure S5. SIV-specific IFN-γ response after immunization heterologous immunization with replicating and non-replicating arenavirus vector (IM). (**A** and **B**), Gag-specific IFN-γ ELISpot responses in individual animals after heterologous immunization with replicating (**A**) and non-replicating (**B**) arenavirus vector platforms. (**C** and **D**), Env-specific IFN-γ ELISpot response in individual animals after heterologous immunization with replicating (**C**) and non-replicating (**D**) arenavirus vector platforms.

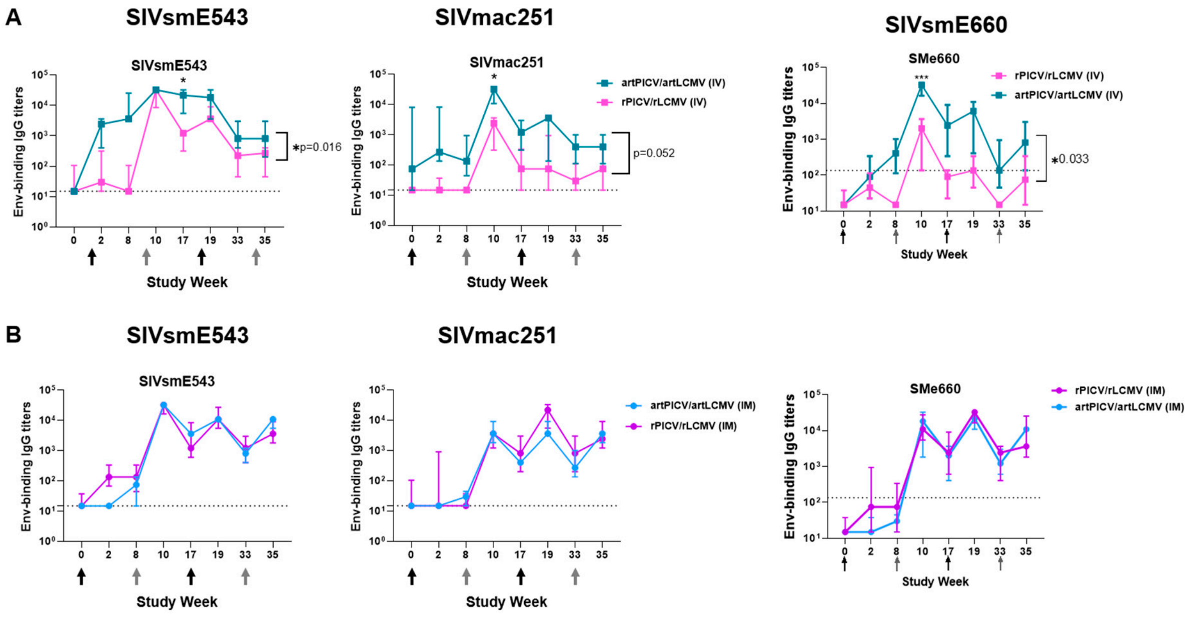

Supplementary Figure S6. Comparison between heterologous immunization of replicating, artPICV/artLCMV and non-replicating arenavirus, rPICV/rLCMV vector for generating SIV envelope binding IgG titers. Binding anti-Env IgG titers against autologous, SMe543 and heterologous, SIVsmE660 and SIV_{mac251} gp120 and after IV (**A**) and IM immunization (**B**). Data represent anti-Env immunoglobulin-G (IgG) endpoint titers over the course of study plotted as median ± IQR (n = 4). Statistical significance for each time point was determined by two-way ANOVA Šidák multiple comparison test (*p=0.01) and for curve comparison, linear mixed-effect model was used (*p=0.016, p=0.052 and *p=0.033, **A**).

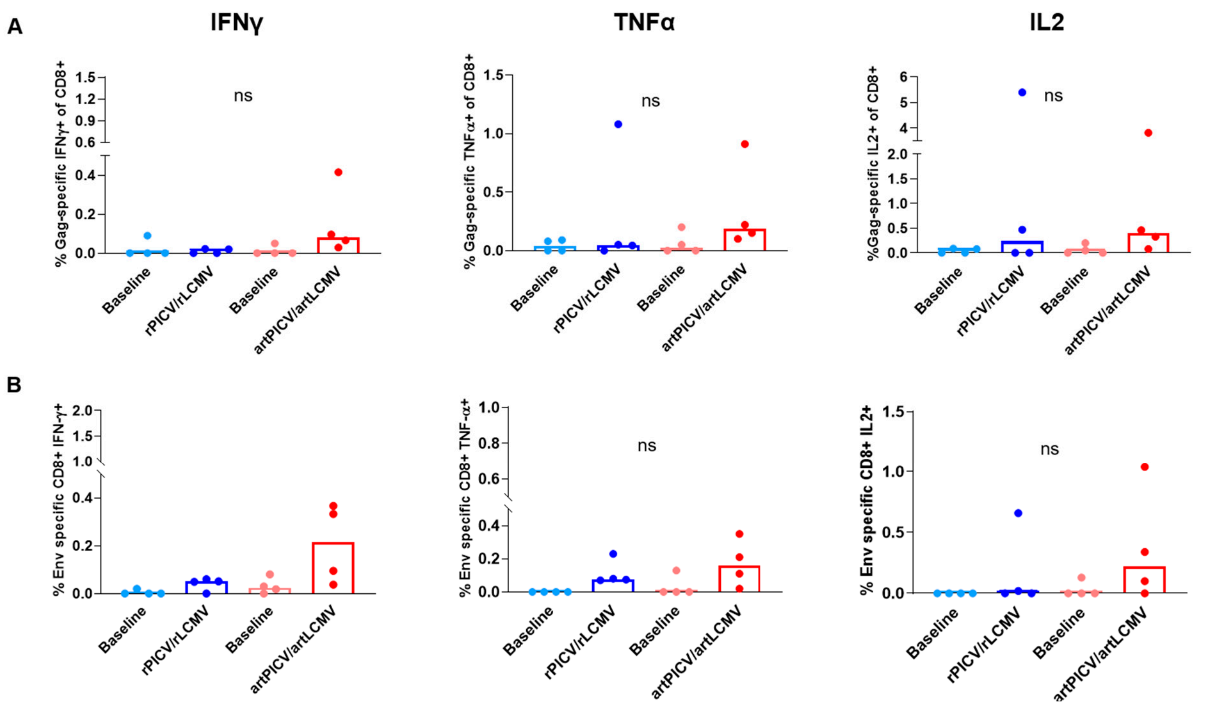

Supplementary Figure S7. Monofunctional CD8 responses. Gag- (**A**) and Env- (**B**) specific monofunctional CD8 T-cell response 2 weeks after immunization with heterologous replicating (artPICV/artLCMV) and non-replicating arenavirus vectors (rPICV/rLCMV). Frequency of IFN-γ+, TNF-α+ and IL2+ CD8+ PBMCs were compared between the two platforms by intracellular staining followed by multiparametric flow cytometry analysis. Data plotted as median ± IQR (n = 4). Statistical analysis was done using non-parametric Kruskal–Wallis test; ns = non-significant. Baseline responses were determined on frozen PBMCs, and 2 weeks after fourth vaccination were evaluated on freshly isolated PBMCs.

CD4 T cells

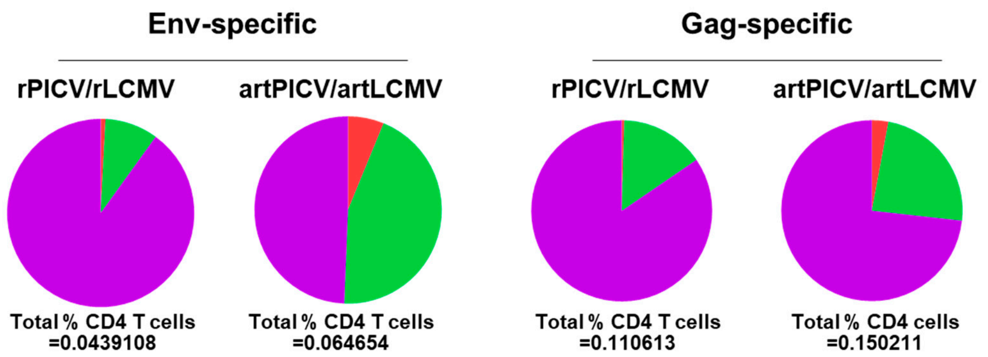

Supplementary Figure S8. SIV-specific (Env ang Gag) CD4 T-cell polyfunctionality after fourth vaccination of non-replicating, rPICV/rLCMV and replicating arenavirus vector, artPICV/artLCMV (IV) in NHPs. Intracellular cytokines evaluated include IFN-γ, IL2 and TNFα in CD4 T cells by multiparametric flow cytometric analysis. Frequency of T cells positive for single (purple color), double (green color) and triple cytokines (red color) are plotted as pie charts.

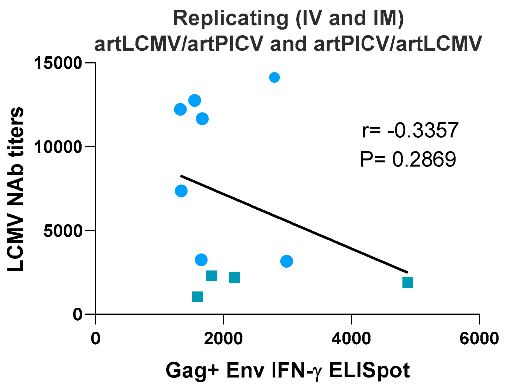

Supplementary Figure S9. Anti-vector immunity. Correlation between SIV-specific IFN-γ ELISpot and LCMV vector nAbs at week 35 (2 weeks after last vaccination dose) for replicating arenavirus vectors. Spearman (*r*) correlation analysis was performed with GraphPad Prism 8.1.2. Round symbols (blue color) represent heterologous IV immunization and square symbols (cyan color) represents IM immunization artPICV/artCMV.

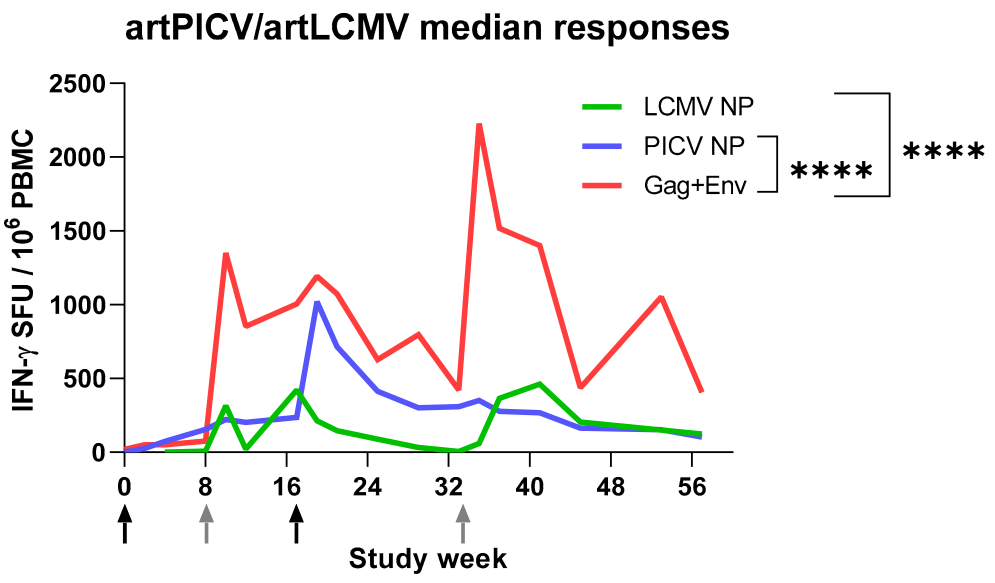

Supplementary Figure S10. Magnitude of SIV-specific IFN-γ responses were higher than vector-specific NP response (IV). IFN-γ ELISpot response to the platform and SIV immunogen was compared for replicating platforms when administered IV as heterologous immunization. Two-way ANOVA, Šidák multiple comparison test, $p<0.0001$ was used for statistical analysis.

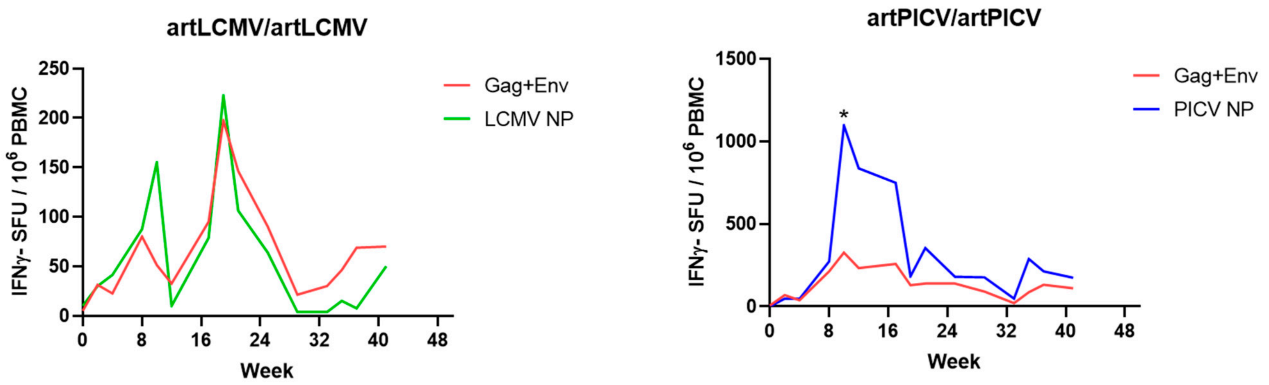

Supplementary Figure S10. SIV and vector NP-specific IFN-γ response elicited by homologous immunization of replicating vectors. Magnitude of SIV and vector NP-specific IFN-γ response elicited by homologous immunization of replicating arenavirus vectors. IFN-γ ELISpot response to the platform and SIV immunogen was compared for replicating vector platforms when administered IV as homologous immunization. Two-way ANOVA, Šidák multiple comparison test, *p<0.0147 was used for statistical analysis.
